# Culturally Adapted STAR-Caregivers Virtual Training and Follow-Up for Latino Caregivers of People Living With Dementia: Single-Arm Pre-Post Mixed Methods Study

**DOI:** 10.2196/66053

**Published:** 2025-06-10

**Authors:** Miguel Angel Mariscal, Celeste Garcia, Lily Zavala, Magaly Ramirez

**Affiliations:** 1 Department of Health Systems and Population Health, School of Public Health, University of Washington, Hans Rosling Center for Population Health, 3980 15th Avenue NE, Fourth Floor, Box 351621, Seattle, WA, 98195, United States, 1 2065439973

**Keywords:** dementia, gerontology, geriatric, older person, aging, Latina, Latino, caregiver, symptom management, digital technology, digital intervention, digital health application, pilot study

## Abstract

**Background:**

Latino caregivers are at an increased risk of negative health outcomes due to the responsibilities of caring for someone with dementia. Although interventions exist to address caregiver burden, they often do not meet the cultural needs of Latino caregivers.

**Objective:**

This study aimed to pilot test the cultural adaptation of the STAR-Caregivers Virtual Training and Follow-Up (STAR-VTF) intervention. The intervention is an evidence-based training program designed to teach family caregivers strategies to manage behavioral and psychological symptoms of dementia (BPSD). Our research team has conducted past studies to identify and perform culturally relevant adaptations to the training modules of STAR-VTF, and this study aimed to pilot these culturally adapted modules with a sample of Latino caregivers.

**Methods:**

Data on feasibility, usability, and acceptability were collected from a pilot test in which Latino caregivers (n=16) used the training modules of the STAR-VTF intervention over a 7-week period. Participants completed usability surveys following the completion of each module, and acceptability was assessed through semistructured interviews (n=14) postintervention. Preliminary outcome measures were also collected, and a descriptive analysis was conducted. The primary outcomes were the Revised Memory and Behavior Problem Checklist (RMBPC) and the Preparedness for Caregiving Scale.

**Results:**

The pilot study results suggest that it is feasible to deliver the culturally adapted STAR-VTF intervention to Latino caregivers, with 94% (15/16) of participants maintaining enrollment through intervention completion. The intervention’s usability was found to be “good” based on an average System Usability Score of 76.7 out of 100 across all training modules. Caregivers were generally satisfied with the training modules. In addition, preliminary outcome results demonstrated a trend of decreased BPSD pre- versus postintervention (RMBPC subscale score: 28.24 to 21.34). Findings also demonstrated decreased caregiver reaction to BPSD pre- versus postintervention (RMBPC subscale score: 40.40 to 37.21) and increased caregiver preparedness based on pre- and postintervention (Preparedness Caregiving Scale score: 1.98 to 2.43).

**Conclusions:**

The pilot study demonstrated that the culturally adapted STAR-VTF intervention is feasible and perceived as easy to use by a small sample of Latino caregivers. We aim to refine the cultural adaptations of the STAR-VTF intervention further based on feedback from study participants. Future studies are necessary to test the efficacy of the intervention and support the broad dissemination of the culturally adapted intervention.

## Introduction

In the United States, Latino populations are disproportionately impacted by Alzheimer disease and related dementias (ADRD). Compared to White adults, Latinos are 1.5 times more likely to have ADRD [1]. By 2060, the number of Latinos with ADRD is expected to grow by 832%, which is the steepest increase in ADRD compared to any other racial or ethnic group, and projections show that by 2060, a total of 3.5 million Latinos will be living with dementia [2,3]. Latinos with dementia primarily rely on family members for their care due to the high cost of formal dementia care, legal status, barriers to accessing dementia care services, language preferences, and cultural preferences [4,5].

Latino caregivers comprise 21% of the estimated 40 million family caregivers in the United States [1]. Caregiving for a person living with dementia can be particularly challenging as ADRD can cause changes in personality and behavior, losses in judgment, orientation, and the ability to understand and communicate effectively [6]. Managing dementia symptoms requires intense supervision and physical support, and compared to other populations, Latino families provide more intense caregiving in terms of level of care and hours [1]. Caregiving for a person living with dementia also places Latino caregivers at increased risk of negative health implications, including high levels of stress, depression symptoms, less engagement in physical activity, sleeping problems, and social isolation [4,7]. Although evidence-based caregiver interventions exist to help address the negative impacts of caregiver burden, they often do not meet the unique cultural needs of Latino families [8]. Due to the negative health implications of dementia caregiving, it is crucial to support Latino caregivers through culturally adapted, evidence-based interventions.

STAR Caregivers (STAR-C) is an evidence-based systematic training program designed to teach family caregivers strategies to reduce behavioral distress and manage behavioral and psychological symptoms of dementia [8]. Caregivers are taught effective communication strategies, activator-behavior-consequence (ABC) behavioral problem-solving strategies, and how to identify and use pleasant activities to reduce behavioral disturbances [9]. STAR-C has been demonstrated to significantly reduce caregiver burden and depression and improve the quality of life of persons with ADRD [10]. Since the original randomized controlled trial of STAR-C in 2005 [11], the program has been adopted in various settings to meet caregivers’ needs. One of the early adoptions of the STAR-C program in a real-world setting was conducted by the Oregon Department of Human Services-State Unit on Aging and Area Agencies of Aging, which served both rural and urban locations in Oregon [9,12]. In this implementation of STAR-C, the training was provided at home by trained professional health consultants to family caregivers. The program has since been adapted to teach caregivers the STAR-C lessons remotely. Tele-STAR, the telehealth-based adaptation of STAR-C, was created to increase access to anyone with a computer and internet connection and to reach rural populations [13]. Tele-STAR used videoconferencing to connect nurse consultants with family caregivers to guide them through the STAR-C lesson plans. The tele-STAR study found that with the transition from in-person training to remote, the family caregiver burden was reduced, and it retained good program and treatment fidelity to STAR-C [13]. Videoconferencing technology has also been used in the Illinois-based CRIS’ Memory Care Program, an adapted STAR-C intervention developed by CRIS Healthy Aging, a nonprofit organization in East Central Illinois that serves older adults primarily in rural communities and of lower income [14]. The latest adaptation of STAR-C in a clinical setting has been STAR-C Virtual Training and Follow-up (STAR-VTF), which was tested at Kaiser Permanente Washington [15]. STAR-VTF is a 6-to-8-week program comprised of six training modules that caregivers complete asynchronously. It also includes 30-minute weekly check-ins with a program coach. Support from program coaches is provided as needed for up to 6 months via secure messaging in the Kaiser Permanente Washington patient portal.

Although STAR-C has been adapted in a variety of ways to expand access to caregivers, it has yet to be tested in large populations of Latino caregivers. Recently, our research team adapted STAR-VTF to improve its cultural relevance and linguistic appropriateness for Latino caregivers [8,15]. We conducted an 8-week pilot study of the culturally adapted training modules from the STAR-VTF intervention. This study reports findings on the feasibility, usability, acceptability, and preliminary outcomes of the pilot study with Latino caregivers. The findings will help improve future iterations of STAR-VTF for Latino caregivers, and more generally, advance our understanding of modifying evidence-based interventions to better serve Latino caregivers of persons with dementia.

## Methods

### Design

**Figure 1.**
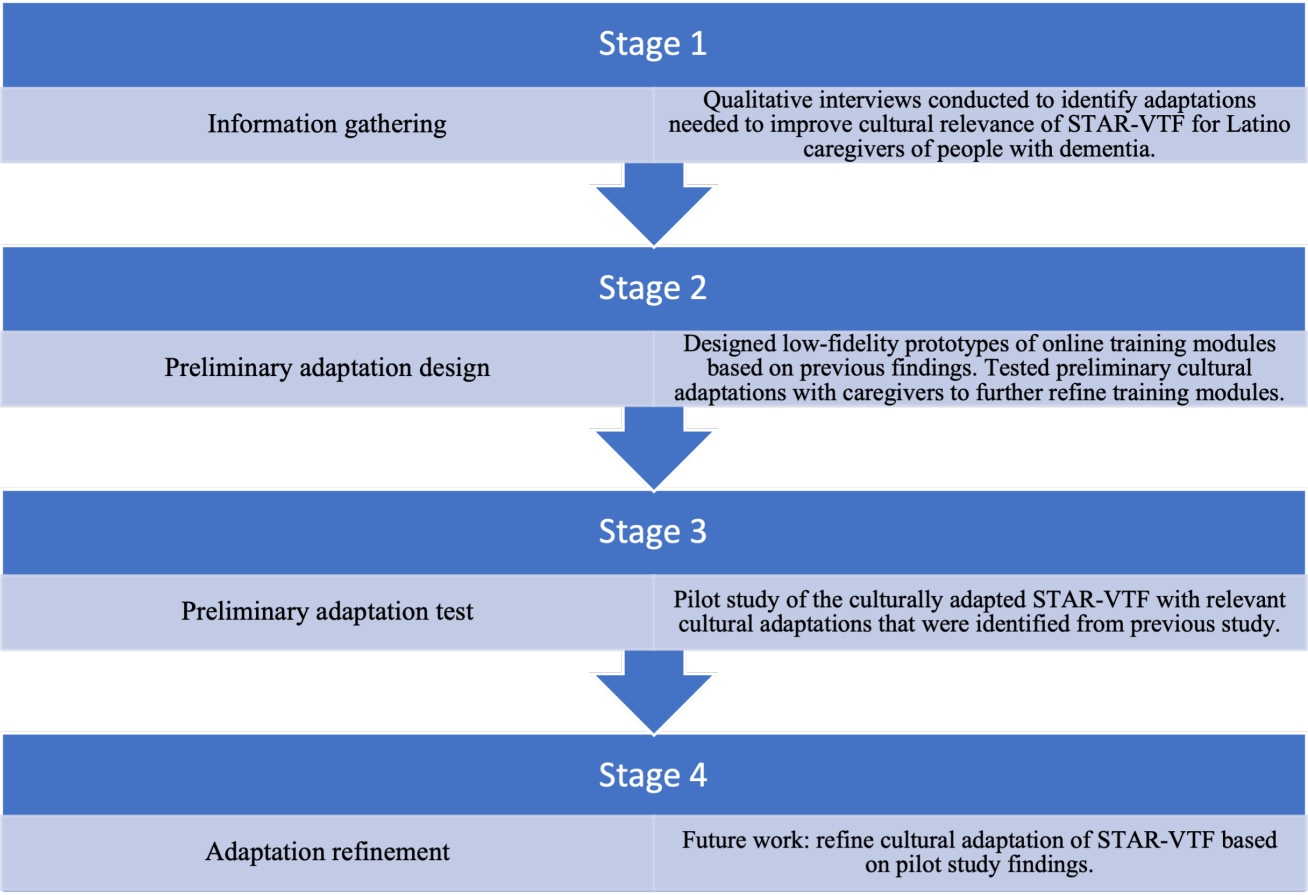
STAR-VTF cultural adaptation process. STAR-VTF: STAR-Caregivers Virtual Training and Follow-Up.

The framework for the cultural adaptation of evidence-based interventions developed by Barrera and Castro [16] guided the methods of this study. The framework includes the following stages in culturally adapting an evidence-based intervention: (1) gathering information to identify ideas about needed adaptations, (2) conducting preliminary adaptations based on these ideas, (3) conducting pilot studies of the preliminary adaptations, and (4) refining adaptations based on results from pilot studies. Our research team has completed the first two stages [8,15]. This study presents findings in the stage 3 process of pilot-testing cultural adaptations (Figure 1).

This single-arm pilot study tested the feasibility, usability, and acceptability of the culturally adapted STAR-VTF training modules. We also trialed data collection procedures and collected preliminary data on persons living with dementia and caregiver outcomes pre- and postintervention to obtain descriptive estimates. Quantitative and qualitative methods were used in this pre- and posttest study.

### Ethical Considerations

The Advarra Institutional Review Board approved this study (Protocol: National Institute on Aging - FY20_Pilot10_Ramirez). If interested, participants were screened for eligibility, and digital or written informed consent was obtained. Consent was collected from participants via a consent form that was provided in English and Spanish, depending on their preference. Translation of the consent form was done by a certified Spanish translator. Participants were provided a US $40 gift card for their participation. All study data were deidentified and stored on secure, password-protected cloud storage.

### Participant Recruitment

**Figure 2.**
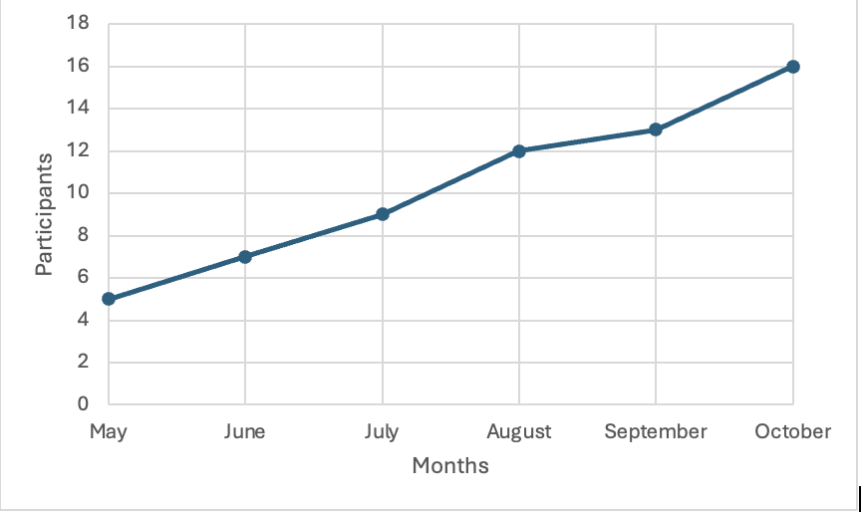
Enrollment timeline.

Potential study participants (ie, Latino caregivers) were identified using the UW Medicine electronic health record, UW Alzheimer’s Disease Research Center Registry, Alzheimer’s Prevention Registry maintained by the Banner Alzheimer’s Institute, and through the distribution of recruitment flyers through professional networks. Potential study participants were contacted by bilingual staff via phone or email to explain the study and answer their questions about study participation. To be eligible to participate, participants had to identify as Hispanic or Latino, be 18 years of age or older, be taking care of a family member or friend with dementia, live with or within 5 miles of the person living with dementia, spend at least 8 hours a week with them, and indicate that the person they care for is experiencing at least three symptoms related to dementia occurring at least three times in the past week. Figure 2 provides additional information on the number of participants who were enrolled over time for this pilot study.

### Intervention: Culturally Adapted STAR-VTF Training Modules

While the full STAR-VTF intervention consists of weekly training modules and weekly 30-minute phone check-ins with a coach [15], this study only pilot-tested the culturally adapted modules. The adaptations used in this pilot study were based on findings from previous qualitative studies [8,15]. These studies interviewed Latino caregivers of persons with dementia, as well as health care and social service providers of older Latinos, to improve the cultural relevance of STAR-VTF. Based on the findings of prior studies, adaptations included revising language viewed as problematic, expanding content to enhance understanding of dementia, and adding cultural examples that reflect family involvement in caring for relatives with dementia and multigenerational living [8,15]. Additional refinements made to the STAR-VTF training modules, after testing preliminary modules with Latino caregivers, included adding empathetic messaging that highlights the importance of viewing the world from the perspective of the person living with dementia, incorporating additional problem-solving examples to demonstrate diverse challenges, and emphasizing caregiver self-care, to ensure caregiver’s mental and emotional health is prioritized [8].

For 7 weeks, caregivers completed training modules asynchronously. They accessed the modules via email or SMS text message. This pilot study was focused on testing the content of the training modules; therefore, caregivers did not receive the coaching component of the STAR-VTF intervention. There were 7 training modules in total. Caregivers were instructed to complete one module per week. The content of the modules is described in Textbox 1.

Textbox 1. **Content of the STAR-Caregivers Virtual Training and Follow-Up training modules.**

**Module 1**

- Provides an understanding and overview of dementia.

**Module 2**

- Introduces caregivers to the behavioral treatment of dementia, realistic expectations, and effective communication.

**Module 3**

- Covers the antecedents, behaviors, consequences (ABC) approach to problem-solving, including rationale and development of an ABC plan. Hypothetical scenarios of how caregivers successfully used the ABC problem-solving to address behaviors (eg, wandering) are included.

**Module 4**

- Instructs caregivers to review the ABC plan and revise it as needed.

**Module 5**

- Covers pleasant events and managing negative thinking.

**Module 6**

- Instructs caregivers to review the ABC plan, pleasant activities schedule, and to revise as needed.

**Module 7**

- Covers coping with caregiving and maintaining the use of caregiving strategies.

The length of the training modules ranged between 7 and 16 minutes each. The modules used text, pictures, and illustrations with a voiceover presentation. Caregivers received the training modules in their preferred language (English or Spanish). They also received a printed or electronic workbook to accompany the lessons (the format depended on their preference). The workbook contained additional educational materials, as well as worksheets for caregivers to write down their ABC plans. Intervention materials, including training modules, voiceovers for modules, and workbooks, were translated into Spanish by a certified Spanish translator. Study participants were enrolled in the study for approximately 8 weeks.

### Measures

#### Feasibility

We assessed feasibility based on National Institutes of Health guidance on appropriate feasibility measures for pilot studies [17]. Feasibility measures included details of the success of procedures to recruit eligible participants, retention, and intervention completion.

#### Usability

To assess usability, we collected data on the System Usability Scale [18] via a REDCap (Research Electronic Data Capture; Vanderbilt University) survey each time a caregiver completed a training module. With the System Usability Scale survey, caregivers rated how easy the training modules were to use. Each item was rated on a 5-point scale ranging from 0=strongly disagree to 5=strongly agree.

#### Acceptability

Assessment of acceptability was accomplished through semistructured qualitative interviews with participants after they completed 7 weeks of the STAR-VTF training modules. Interview questions explored the participant’s experience with the program and solicited feedback on recommendations for further improving the program.

#### Person Living With Dementia and Caregiver Outcomes

The primary outcomes were the Revised Memory and Behavior Problem Checklist (RMBPC) [19,20] and the Preparedness for Caregiving Scale [21]. The outcomes were collected at baseline and 8 weeks postenrollment via a REDCap survey. For the RMBPC, caregivers rated memory, depression, and disruptive behavior problems in the person living with dementia. The instrument contains 24 items (7 memory-related, 8 depressive, and 9 disruptive) that assess problem behaviors and are rated for frequency of occurrence during the past week and caregiver reaction, which refers to the extent to which caregivers were distressed by the problem behaviors. The Preparedness for Caregiving Scale was used for caregivers to rate how prepared they are for various aspects of caregiving. The instrument contains 8 items that ask caregivers how well prepared they believe they are to provide physical care and emotional support, deal with the stress of caregiving, and set up in-home support services. Each item is rated on a 5-point scale ranging from 0=not at all prepared to 5=very well prepared.

### Data Collection

#### Timeline of Data Collection

**Figure 3.**
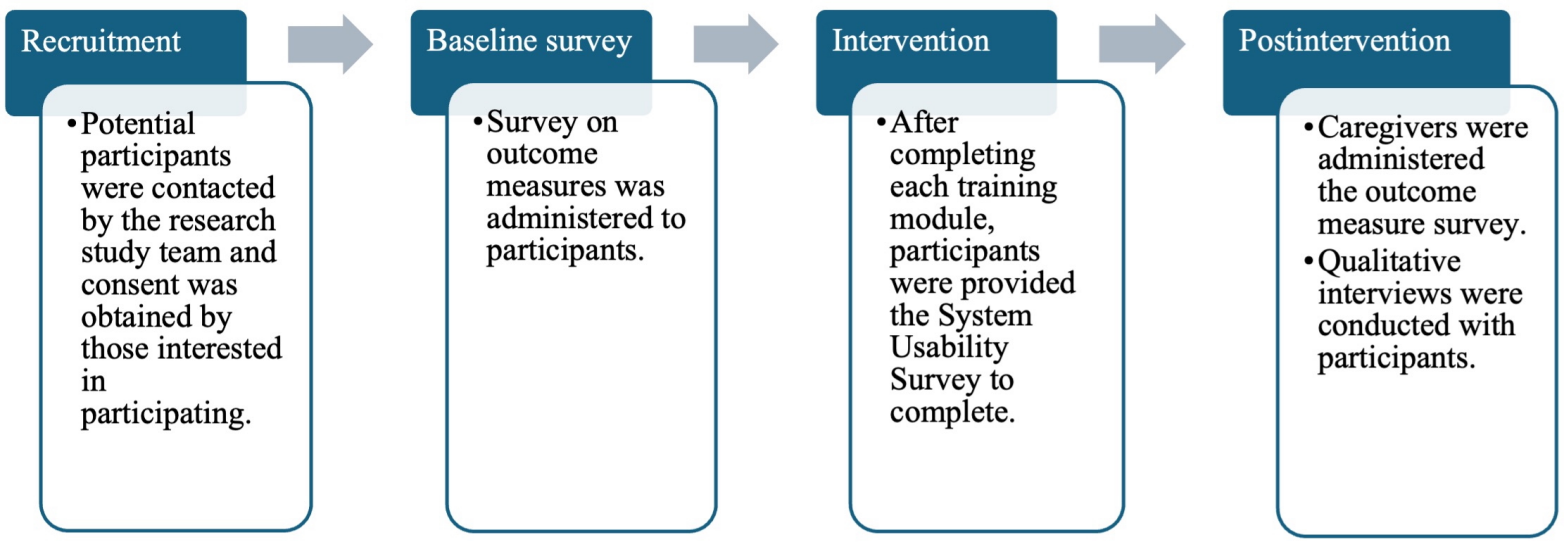
Time of data collection.

The data for this study were collected pre- and postintervention and throughout the intervention period. For reference to the data collection timeline, see Figure 3.

#### Quantitative Data Collection

We collected outcomes data on the RMBPC, Preparedness for Caregiving Scale, and System Usability Scale via REDCap surveys in Spanish and English. The Spanish versions of these scales have been validated and found reliable [20,22,23]. Research assistants on the project delivered a link to the surveys via email or SMS text message to caregivers. These surveys were provided to participants at baseline and postcompletion of the training modules.

#### Qualitative Data Collection

All participants who reached the end of the study were invited to participate in a semistructured interview conducted in Spanish or English. All interviews with participants were conducted over the phone with bilingual research team members (CG and LZ). The interview guide included questions about their opinions on the program training components, suggestions for improving course content and design, and the quality of the Spanish translation of training modules (Multimedia Appendix 1). The average length of interviews was approximately 30 minutes. Interviews with participants were audio recorded, and the transcription of the interviews was done by a professional transcription service.

### Quantitative Data Analysis

We analyzed quantitative data using descriptive statistics. Based on National Institutes of Health guidance, we did not conduct outcome analysis for effect size estimates since an effect size estimated from a pilot study is inherently unstable and would not provide helpful estimation for power calculations [17].

In the descriptive analysis, we only included study participants who completed the outcome assessments at baseline and 8 weeks. If study participants had missing values in their responses (either because they skipped an item or responded with “Don’t Know/Not Applicable”), then we imputed their missing values. The missing data were imputed by taking the highest possible score that participants could obtain if they answered all items and then multiplying that score by the quotient of the participant’s actual score divided by the highest possible score they could have received from the items they answered.

### Qualitative Data Analysis

To analyze qualitative data, we used qualitative analysis methods outlined by Miles et al [24]. Interview transcripts were coded and analyzed in their original language (English or Spanish) using Dedoose (version 9.2.007), a cloud app for managing, analyzing, and presenting qualitative and mixed method research data. First, bilingual team members (MAM, CG, and MR) read the transcripts and wrote brief notes of what was in the data. Next, the first (MAM) and second (CG) authors independently coded transcripts using inductive codes. They convened weekly with the senior author (MR) to discuss the application of codes, refine the codebook consisting of inductive codes, and reach a consensus on coding discrepancies. After coding three transcripts, the codebook was comprehensive, and a few additional codes needed to be added. The first author individually coded the remaining transcripts, with guidance from the senior author as needed. Descriptive and in vivo codes were used in the coding process.

The first author identified preliminary themes based on the inductive codes by collating and reviewing excerpts of each code. During this process, relationships among codes and relationships within and among themes were assessed. A collection of relevant excerpts for each theme was pulled from the data while reviewing coded excerpts. Recommended areas of assessment in the third stage of Barrera and Castro’s [16] framework on cultural adaptations of evidence-based interventions, such as satisfaction with intervention elements and suggestions for improvement, were used to help guide theme creation. The preliminary themes were iteratively refined with assistance from the second and third authors (CG and MR) to ensure that subthemes within the themes were relevant to the parent theme and that the final themes were distinguishable.

## Results

### Participant Characteristics

In total, 16 participants enrolled in the pilot study of the culturally adapted STAR-VTF training modules. Table 1 provides demographic characteristics of the study participants. Demographic characteristics were collected at baseline.

**Table 1.** Participant characteristics.

Characteristics	Study participants (n=16)
Age, mean (SD)	51.2 (13.2)
Gender, n (%)	
Man	4 (25)
Woman	12 (75)
Hispanic or Latino origin, n (%)	
Mexican, Mexican American, Chicano or Chicana	10 (62)
Puerto Rican	1 (6)
Other	5 (31)
Race, n (%)	
Black or African American	1 (6)
White	11 (69)
Other	3 (19)
Prefer not to answer	1 (6)
Highest level of education completed, n (%)	
Primary level education	1 (6)
Secondary level education or GED^a^ or equivalent	2 (12)
Some college	2 (12)
Associate’s degree	2 (12)
Trade or vocational training	1 (6)
Bachelor’s degree or higher	8 (50)
Household income before taxes (US $), n (%)	
Less than 15,000	1 (6)
Between 15,000 and 19,999	1 (6)
Between 20,000 and 24,999	1 (6)
Between 25,000 and 34,999	1 (6)
Between 35,000 and 49,999	2 (12)
Between 50,000 and 74,999	2 (12)
100,000 or above	6 (37)
Prefer not to answer	2 (12)
Current occupational status, n (%)	
Employed	9 (56)
Unemployed	3 (19)
Stay-at-home caregiver	2 (12)
Retired	2 (12)
Current marital status, n (%)	
Married	12 (75)
Divorced	1 (6)
Separated	1 (6)
Never married	2 (12)
Primary language spoken at home, n (%)	
English	8 (50)
Spanish	8 (50)
Received STAR-VTF intervention materials in Spanish, n (%)	7 (44)
Caregiver’s health insurance plan, n (%)	
Insurance through employer	8 (50)
Insurance purchased directly	1 (6)
Medicare	3 (19)
Medicaid	1 (6)
No Insurance	3 (19)
Person living with dementia health insurance plan, n (%)	
Insurance purchased directly	1 (6)
Medicare	9 (56)
Medicaid	5 (31)
TRICARE or military health care	1 (6)
Persons living with caregiver, n (%)^b^	
Spouse or partner	12 (75)
Children or grandchildren older than 18 years of age	5 (31)
Children or grandchildren younger than 18 years of age	4 (25)
Relatives (besides spouse or partner and children or grandchildren)	7 (44)
Other	1 (6)
Household size, mean (SD)	3.4 (1.5)
Devices owned, n (%)^b^	
Smartphone	15 (94)
Tablet	11 (69)
Laptop	11 (69)
Computer	7 (44)
Internet access, n (%)	
Cellular data plan	5 (31)
Broadband internet	10 (62)
Satellite internet	1 (6)
Caregiver’s relationship to the person living with dementia, n (%)	
Spouse or partner	3 (19)
Adult child	9 (56)
Other close family member	3 (19)
Friend or other nonrelative	1 (6)
Gender of person living with dementia, n (%)	
Man	4 (25)
Woman	12 (75)
Lives with person living with dementia, n (%)	10 (62)
Years in the caregiving role, mean (SD)	4.3 (3.28)
Caregiving hours per week, mean (SD)	60.2 (50.9)

a GED: General Educational Development.

b Column percent may not add up to 100% because categories are not mutually exclusive.

### Feasibility

We achieved satisfactory feasibility based on recruitment, retention, and intervention completion measures. We recruited 16 study participants. The initial recruitment goal was to recruit 20 participants, and we reached 80% of our goal (16/20). Of the participants recruited, 94% (15/16) of participants were retained for all 8 weeks of the pilot study. The proportion of participants who completed all 7 training modules and participated in all study procedures (qualitative exit interviews, pre- and postoutcome assessments, and weekly usability surveys) was 75% (12/16).

### Usability

Usability was assessed for each training module. Average scores and SDs for each module were calculated (Table 2). The average System Usability Score across all 7 modules is 76.7 out of 100, which is considered “good” usability, indicating that caregivers perceive the STAR-VTF training modules as easy to use [24,25].

**Table 2.** System Usability Scores.

Week	System Usability Score, mean (SD)
1 (n=13)	77.8 (10.8)
2 (n=12)	77.7 (12.9)
3 (n=10)	74.6 (19.2)
4 (n=13)	73.7 (16.3)
5 (n=12)	75.8 (14.3)
6 (n=9)	79.2 (14.5)
7 (n=11)	78.0 (13.7)

### Acceptability

The results of the semistructured interviews provided information about the cultural adaptations of the STAR-VTF training modules. The qualitative results also provide a further understanding of what changes are needed to revise the cultural adaptations further.

### Theme 1: Latino Caregivers Were Satisfied With the STAR-C Intervention Content and Design Elements of the Training Modules and Accompanying Workbook

#### Overview

Latino caregivers were generally satisfied with the content covered in the STAR-C intervention, including components on dementia education, ABC problem-solving, and caregiver support strategies. They also expressed satisfaction with the design of the training modules and the accompanying workbook.

#### Dementia Education Component

Latino caregivers found the dementia education information beneficial in understanding their relatives’ dementia condition and how it can progress. There was also a consensus among most caregivers that they liked the empathetic messaging embedded within the dementia education component. Caregivers felt that it was helpful to be reminded that their family member has a condition of the brain and that some of the things their family member says or does are not on purpose, and to be encouraged to have patience and understanding. A caregiver providing care to their father said the following about the messages reminding caregivers to be empathetic:

*And I felt like it was an important reminder because I guess just like with anything else, even as a parent of young children, I get caught up in the day-to-day and the getting things done, and there’s deadlines, and there’s work and home life and all sorts of things. And so it is nice to have that reminder that it’s not just ... not to take things personally and not to be so quick to react. But just a gentle reminder, there may be another reason why my dad is saying something or not doing something. And yeah, just not to take it personally, so that was a helpful reminder*.

[ID 11]

#### ABC Problem-Solving Component

Many Latino caregivers expressed that the ABC problem-solving strategy of the training was helpful and found the examples used to demonstrate how to implement the strategy to be relatable to their own caregiving experience. One of the training modules demonstrated a hypothetical caregiver using the ABC problem-solving strategy to address a person living with dementia wandering. When asked about their thoughts on this example, one of the caregivers stated:

*So I thought it was very relatable. And then sort of just kind of showcasing what happened, I think, made it relatable in the sense that you can sort of put yourself in that situation. And then for me, I was like, “Okay, yeah. I could see how this happens, and then what can you do to utilize those ABCs to then basically make it useful for your own personal experience in a similar situation*.”

[ID 14]

Latino caregivers also shared how the ABC problem-solving strategy was successful in helping them manage their relative’s behavioral and psychological symptoms of dementia and provided anecdotes of their experience applying the ABC problem-solving strategy. For example, one of the caregivers described how they used the strategy to address their mother’s experience of washing dishes. The caregiver said:

*So my mom would get really upset and would complain of dizziness around the same time every day. And I thought that was odd. And then when I was doing this and I took the ABC, I realized that the reason my mom was getting upset was because she would wash the dishes at the same time every day at, and sometimes I would go by and tell her, “Oh, turn the water higher,” or, “Oh, put more soap.” And I think me trying to coach her was upsetting her because it made her feel like she didn’t know what she was doing. So she would go, and she would say she’s dizzy, and she would start to cry. And then because I noticed the timing, what happened before and after, I stopped doing that. I just let her wash the dishes.*

[ID 27]

#### Caregiver Support Strategies Component

Latino caregivers overwhelmingly related to the caregiver support strategies, encouraging caregivers to practice self-care. Caregivers expressed the importance of caring for themselves to care for others. They also acknowledged the importance of taking time for themselves. For example, a Spanish-speaking caregiver providing care to his wife explained the reasons why it was important for caregivers to prioritize their own self-care.

*It’s good that you made us see what we need, also we need to take care of ourselves to be able to provide care. In my case, my wife needed me to be good so I could take care of her well. One needs to sleep well and eat well to give the best help to the sick*. (translated quote)

[ID 06]

#### Design of Training Modules and Accompanying Workbook

Latino caregivers were satisfied with the design of the training modules and the accompanying workbook. Some caregivers said they liked the pacing of the training modules and the images used. One caregiver shared the following about the training modules:

*I like the pace of it, and I liked the way that it was almost like going through a PowerPoint presentation. I liked that there were slides that kind of bulleted the info that I could either read along to or that it included images that were ... let me see. Images of people of color and not just an elderly White family*.

[ID 11]

Some of the caregivers described the workbook as a helpful resource to refer to information and strategies that had been covered in the training modules. For example, one of the caregivers planned to continue referencing the workbook after the research study ended. They stated:

*So I think for me it’s [the workbook] going to be a helpful guide now that I don’t have access to the videos to go through it and remind me of things and I can share it with my other family members who are also trying to help with caring with my mom. So I feel like the workbook is kind of the legacy of those classes*.

[ID 27]

### Theme 2: Suggestions for Improving Program Content and Making Training More Engaging

#### Overview

Although Latino caregivers were generally satisfied with the training modules, some caregivers shared suggestions that they felt would enhance the intervention. The areas for improvement that Latino caregivers suggested are the inclusion of information on additional resources and improving the interactivity of training modules to make videos more engaging.

#### Including Information on Additional Resources

Latino caregivers expressed wanting additional information on resources outside of the program that they can access to help support people living with dementia. Although some resources were presented in the training modules, caregivers shared they would like more resources on dementia care and community organizations serving people living with dementia and their families. For example, one caregiver shared:

*Maybe what might be helpful is if you had ... now this would be just ... I guess you’d have to make it unique for every state, but maybe give them some resources of where they might be able to reach out to. There’s different Medicaid programs that might be eligible for. There might be local dementia associations that they could contact, those types of things, right? So resources that people might be able to reach out to, especially ones that offer services in Spanish.*

[ID 17]

#### Improve Interactivity to Make Training Modules More Engaging

Some Latino caregivers provided suggestions for improving the interactivity of training modules to make learning program content more engaging. A suggestion to improve the interactivity of program content was to include knowledge checks or short quizzes in which caregivers can test their knowledge of the material covered. As one caregiver shared:

*Having something that’s a little more interactive where it’s not just a video, but almost like a course where you watch a video and then ... having both options like an actual paper workbook or having the option to select multiple choice or just to keep the viewer engaged in the video. So even if it’s like, “Here’s a summary. Which of these apply?” Select A, B, C, or D, all of the above, or something like that, just so that it really just keeps the viewer engaged*.

[ID 11]

Latino caregivers also felt there were areas in which the training modules could be improved visually to make them more engaging. A suggestion that was shared by caregivers was to include video reenactments to demonstrate how strategies learned can be used in real-world situations. For example, one caregiver shared they liked the ABC strategy examples in the training module but mentioned that if they were recreated in a video depicting a role-play of the scenario, it would be more engaging. As the caregiver explains:

*I feel like an actual video example of the dialogue would go a lot further than the pictures with the text-next-to-them pictures. If I remember correctly, that’s what they were. They were pictures of the humans, of the woman and the person, her caregiver, and then there was just dialogue text next to them. It made sense. It just seems very dry. It seemed very dry. And I didn’t really feel like I was paying attention as much as I should have in those moments*.

[ID 24]

Some caregivers also suggested the importance of including testimonials from fellow caregivers of patients with dementia about their experiences with caregiving and how they applied the STAR-VTF training. One caregiver shared how incorporating caregiver testimonials can help demonstrate how to implement strategies covered in the training, such as the importance of caregiver self-care:

*I think probably maybe interviewing some caregivers and having them share their journey from not knowing what’s going on with your mother ... she’s crazy ... to understanding what’s happening and the realization that it is an illness and examples of how they continued on with taking care of their health. It’s very stressful. It’s very stressful. I mean, it creates a physical response in your own body. And so I think it’s just the affirmation from others that what they’ve done to manage their own time and their own feelings and examples like that and reassuring us that it’s okay. It’s okay to leave them and do something fun like go to a movie with your husband instead of feeling guilty about it.*

[ID 28]

### Theme 3: Caregivers Share Dementia Care Information With Others and Suggest Outreach Strategies

#### Overview

Latino caregivers shared how they have increased awareness of dementia care strategies by sharing the information they learned in the training modules with relatives. Caregivers generally shared program content by word of mouth or by sharing the link to the training modules that were provided to them. Latino caregivers suggested additional avenues to spread awareness of the STAR-VTF program, such as working with community partners and using social media.

#### Sharing Information With Relatives

Multiple Latino caregivers shared that they valued the information provided in the training and that they shared this information with family members who assist in the care of the person living with dementia and with family members who are in a similar caregiving role. For example, one caregiver said:

*So I shared the videos with a group chat for the seven-week period. It was a group chat of my caregivers for my Nana, including myself. So it’s going to be my sisters, my two sisters, my two cousins, my father, my uncle, and my aunt. And all of us took part in that group chat, and I shared it with them every time I got a new video because I felt like it was something that we could all benefit from. We all share the same struggle right now. And even though we’re not in the same place, as far as mentally, taking care of my grandmother, this is a step to getting us all on the same page. And it seemed like the right thing to do to share the information that I was getting and make sure that my family understood why I was doing what I was doing.*

[ID 24]

#### Community Outreach Strategies

To further improve the STAR-VTF program outreach, some caregivers shared that it is important to partner with trusted Latino community organizations, community health workers, religious centers, and caregiving organizations. As one caregiver explains, to reach Latino communities, it is important to consider partners who have established trust in the community to establish the credibility of the program. The caregiver said:

*So often Latinos they feel more trusted with people that they know, so family and friends or church or organizations. So if you can tap into those kind of communities to share it, I think, will probably be your best bet in getting the word out. Even my mom, she’s very hesitant to go to the doctor. I have to take her because she grew up in a generation where they kind of mistrust doctors, but they trust their sisters or cousins. And if they come at them with information, they’re more apt to use it than if it comes from a really formal setting. So to say that word of mouth, that community-centered family groups, community groups, churches, any kind of groups that have a good connection to the Latino community, I think they’re more apt to use the information you’re providing*.

[ID 27]

Some caregivers also suggested advertising the intervention in medical facilities and partnering with health professionals. As one Latino caregiver shared:

*Working with the community health workers, they can put it out (information on intervention) or maybe have something in the rural area at the doctors. I know they have a lot of doctors. So if it’s just one doctor’s office, if you have some handout or a little small TV screen that’s on loop playing some of that information, it would be good*.

[ID 09]

Some Latino caregivers also shared the importance of the STAR-VTF program building a digital presence by posting the training modules on social media, such as Facebook, to reach Latino populations. Caregivers believed delivering the STAR-VTF program through these platforms would increase the reach of the program. For example, one caregiver shared:

*You can put them (training videos) on Facebook, on social media, and then when you’re on social media, you could pop up and ... because you could be on social media on the weekends, and then your study would pop up, or your videos would pop up on the weekends and ... because people are always on social media day, morning, night, weekends, holidays, so if you had your content available on social media, then they would be able to view it*.

[ID 08]

### Preliminary Outcomes

The average RMBPC score for a person living with dementia behavior problems changed from 28.24 (SD 18.31) at baseline to 21.34 (SD 16.76) postintervention, indicating a decreased frequency of behavior problems (Table 3). The average RMBPC score for the caregiver’s reaction to behavior problems changed from 40.40 (SD 16.68) at baseline to 37.21 (SD 14.60) postintervention, indicating decreased caregiver reaction to behavior problems. Furthermore, the average Preparedness for Caregiving Scale score changed from 1.98 (SD 0.33) at baseline to 2.43 (SD 0.32) postintervention, indicating increased preparedness for caregiving. The findings show a trend toward decreased behavior problems in the person living with dementia, decreased caregiver reaction to behavior problems, and increased caregiver preparedness after versus before the intervention.

**Table 3.** Results for RMBPC^a^ and Preparedness for Caregiving Scale at weeks 0 and 8.

	Week 0, mean (SD)	Week 8, mean (SD)
RMBPC		
Problem Frequency, overall (n=14)	28.24 (18.31)	21.34 (16.76)
Problem Frequency, memory subscale (n=14)	7.70 (5.35)	4.98 (3.63)
Problem Frequency, disruption subscale (n=14)	10.24 (7.95)	6.39 (6.20)
Problem Frequency, depression subscale (n=14)	10.73 (10.36)	9.23 (10.35)
Caregiver Reaction, overall (n=13)	40.40 (16.68)	37.21 (14.60)
Caregiver Reaction, memory subscale (n=13)	19.21 (5.70)	18.64 (6.52)
Caregiver Reaction, disruption subscale (n=13)	9.35 (6.54)	8.14 (5.27)
Caregiver Reaction, depression subscale (n=13)	11.85 (7.52)	10.43 (5.88)
Preparedness for Caregiving Scale Score (n=14)	1.98 (0.33)	2.43 (0.32)

a RMBPC: Revised Memory and Behavior Problem Checklist.

## Discussion

### Principal Results

This study aimed to pilot-test the culturally adapted STAR-VTF training modules among Latino caregivers of people living with dementia. Delivering the culturally adapted STAR-VTF training modules to Latino caregivers was feasible, with a high proportion of participants completing the study procedures. The training modules were also found to be easy for Latino caregivers to use based on the high average of System Usability Scale scores across modules.

The qualitative analysis of interviews with Latino caregivers who completed the 8-week program revealed their high satisfaction and acceptability with the training modules and components on dementia education, ABC problem solving, and caregiver support strategies. The study also identified areas for improvement in the training modules, such as providing caregivers with information on additional resources and enhancing the engagement of training videos. The study also provided valuable insights into expanding the intervention’s reach, including through word of mouth, partnerships with community organizations, and social media. These findings will significantly contribute to the refinement of the culturally adapted STAR-VTF training modules for Latino caregivers. The culturally adapted STAR-VTF intervention was also found to have positive changes in the outcomes of behavioral problems among people living with dementia and caregiver preparedness among the small sample of Latino caregiver participants. Further research is needed to examine the efficacy of the culturally adapted STAR-VTF intervention.

### Comparison With Prior Work

This is the first pilot study that tests a cultural adaptation of the STAR-VTF evidence-based intervention for Latino caregivers. Our findings on the feasibility of the culturally adapted STAR-VTF intervention are comparable to other interventions for dementia caregivers delivered digitally, which were also feasible [26,27]. However, unlike these studies, this study consists exclusively of Latino participants, which provides further information on the feasibility of digital interventions for Latino caregivers. Preliminary findings of positive mental health outcomes and improvements in caregiver preparedness are similar to findings from a systematic review of literature on internet-based interventions aimed at supporting family caregivers of people living with dementia [28]. The studies presented in the systematic review of digital dementia caregiving interventions reported reductions in caregiver stress, depression, and strain and improvements in self-efficacy [28]. However, conclusions from these studies and this pilot study should be made with caution due to study design limitations, as these studies consist of quasi-experimental, small randomized controlled trials, pre-post studies, and feasibility studies.

Our findings on acceptability and Latino caregivers’ satisfaction with training components in this pilot study were consistent with the identified needs and wants of caregivers who participated in a qualitative study to test low-fidelity versions of the culturally adapted training modules [8]. In the pilot study, Latino caregivers expressed how the module on dementia education was especially beneficial to their understanding of dementia; this finding aligned with caregivers’ perspectives of wanting more dementia education in the previous study of low-fidelity training modules [8]. The desire to learn about ADRD from Latino caregivers of people living with dementia is also consistent with findings from a qualitative study that examined barriers and facilitators to increasing engagement of Hispanics or Latinos in clinical trials on ADRD [29]. In this study, a majority of caregivers expressed an eagerness to receive information about ADRD, including details on the different types of ADRD, specific risk factors for Latino communities, and hereditary characteristics [29].

Another aspect of the training modules that resonated with Latino caregivers, which was highlighted in our previous qualitative study, was the inclusion of reminders for caregivers to be empathetic toward people living with dementia. Our findings on caregiver satisfaction with the caregiver support strategy component are also similar to findings from our previous study in which caregivers highlighted the importance of self-care [8]. The need for information on self-care by Latino caregivers was also highlighted in a qualitative study that explored the needs of Latino caregivers of relatives with early-onset Alzheimer disease [30]. This study found that among their 27 participants, they overwhelmingly expressed the need for additional information concerning self-care, especially mental health care strategies and stress management [28]. Caregivers’ high satisfaction with the training components piloted in this study suggests that we effectively integrated feedback from Latino caregivers to meet their needs in the training content.

Regarding our findings on areas of improvement, Latino caregivers’ needs for additional dementia care–related resources are consistent with findings from a mixed methods study on the health information–seeking behavior of Latino caregivers of people living with dementia. The study on health-seeking behaviors found that Latino caregivers desire to seek various types of dementia information, including the availability of community resources, health care services, and treatment [31]. Caregivers in this pilot study also suggested improving training videos by making them more engaging with video reenactments of caregiving scenarios, knowledge checks, and caregiver testimonials. This aligns with findings from our prior qualitative study wherein Latino caregivers who viewed the low-fidelity modules expressed the idea of having real people in the videos to depict caregiver scenario examples [8]. Multiple studies have found that using video vignettes in the form of telenovelas and Spanish soap operas is an acceptable and effective method of delivering health information to Latino caregivers [32-34]. Furthermore, a study that evaluated the acceptability of a web-based course for caregivers of older adults found that caregivers liked having postmodule quizzes, and they also had a caregiver suggest the use of caregiver testimonials in course modules [35]. Our findings show that these preferences in web-based training are also warranted among a sample of Latino caregivers.

### Limitations

Although preliminary outcomes were positive, this study had several limitations. First, due to the small budget, we were not able to pilot-test the full STAR-VTF intervention, which consists of weekly training modules and weekly 30-minute phone check-ins with a coach. It is during these check-ins that coaches review caregivers’ ABC plans. Future pilot studies will include the coaching component, which will also ensure that the ABC plans are being developed appropriately. Second, general limitations of pilot studies apply, such as the inability to determine efficacy and estimate effect sizes for power calculations of a larger-scale study [17]. The target sample size of 20 for this pilot study was determined based on logistical constraints, as it was the maximum feasible within the available budget and study timeline. Third, the pre-poststudy design is subject to limitations due to the lack of a comparison group, which relies on conclusions formed from the temporal relationship of the measurements to the intervention. This type of study does not allow researchers to control for other changes that could have occurred around the same time as the intervention that could have influenced outcome results [36]. A limitation of the qualitative portion of this study is that caregivers had trouble recalling details about earlier training modules. A caregiver shared that since it had been over a month since they viewed the first couple of training modules, it was difficult to remember some of the specific details of the content covered. When conducting interviews with participants, the research assistants (CG and LZ) would sometimes need to remind caregivers about earlier training modules. The time gap between viewing the earlier training modules and being interviewed can result in recall bias, and participants may have omitted important details regarding their experience. Another limitation of this study is that 10 of the 14 interviews were conducted by the same research assistant who recorded the voiceover for the modules. If participants recognized that it was the same person, it may have led to social desirability bias, as participants may have provided answers that would be viewed favorably by the research assistant.

### Conclusions

This pilot study found the culturally adapted STAR-VTF intervention to have high levels of feasibility, usability, and acceptability. In general, caregivers found the training modules beneficial and relatable and were highly satisfied with the cultural adaptations to the STAR-VTF intervention. The feedback from the qualitative interviews with Latino caregivers will help inform decisions during the fourth stage (Adaptation refinement) of the Barrera and Castro [16] framework on the cultural adaptation of evidence-based interventions to refine future iterations and meet Latino caregiver needs. Although future research is still necessary to test the efficacy of the culturally adapted STAR-VTF program, this pilot study provided valuable information about the feasibility, usability, and caregiver experiences with the intervention. This study also contributes to the broader knowledge of modifying evidence-based interventions to better serve Latino caregivers of persons with dementia.

## Supplementary material

10.2196/66053 Multimedia Appendix 1 English and Spanish interview guides.

## Abbreviations

ABC: activator-behavior-consequence
ADRD: Alzheimer and related dementia
REDCap: Research Electronic Data Capture
RMBPC: Revised Memory Behavior Problem Checklist
STAR-C: STAR Caregivers
STAR-VTF: STAR Virtual Training Follow Up
