# Supplementary material for: Culturally Adapted STAR-Caregivers Virtual Training and Follow-Up for Latino Caregivers of People Living With Dementia: Single-Arm Pre-Post Mixed Methods Study
Source: JMIR Aging. 2025 Jun 10;8:e66053. doi: 10.2196/66053 (PMC12172800; doi:10.2196/66053)
Supplement: Multimedia Appendix 1 [file aging-v8-e66053-s001.docx]

**Interview Guide**

*****Start recording*****

**Introduction**

*Before we begin, I’d like to remind you that your participation in this study is completely voluntary. You can choose at any point to stop the interview.*

*As part of the study, you used the CUIDA program for the past 2 months. The program involved 7 videos and a workbook. The program was designed to help family caregivers, like yourself, learn how to manage symptoms of dementia.*

*As part of the study, you were also asked to complete surveys online.*

*During this interview, I want to ask you about your experience with the CUIDA program and with completing surveys online. We ask that you provide your honest opinion because it will help us make the program better for family caregivers who use it in the future. We are very interested in your thoughts on how we can make the CUIDA program better for Latino families.*

*With that said, do you have any questions before we begin?*

**General Questions**

*I’ll start by asking general questions about the CUIDA program.*

1. What do think about the program?
   1. Probe: How helpful were the videos for you?
   2. Probe: How helpful was the workbook for you?
2. What did you like about the program?
   1. What did you like most about the videos?
   2. What did you like most about the workbook?
3. Was there anything missing from the program that you would have liked to see?
   1. How do you think we could make the videos better in the future?
   2. How do you think we could make the workbook better in the future?
4. [If caregiver received intervention in Spanish]: What did you think about the Spanish language in the intervention?
   1. How accurate was the Spanish?
   2. How easy or hard was it to understand the Spanish?
   3. What suggestions do you have for us to improve the CUIDA program in Spanish?

**Dementia Education**

*You might recall that the first video in the program provided information about dementia. It talked about what dementia is, the stages of dementia, and how as dementia progresses, people are less able to control their behavior.*

1. What did you think about this first video?
   1. What information did you find particularly helpful?
   2. What other topics about dementia do you wish would have been discussed?

**Empathy**

*Some caregivers have said that they like to be reminded that their family member has a condition of the brain, that some of the things their family member says or does are not on purpose, and for the caregiver to have more patience.*

1. Do you relate to this? Why or why not?
2. Do you recall times when the videos had these types of reminders? For example, one of the videos said, “It’s important to remember that the changes happening to your family member are not on purpose.”
   1. If yes: What did you think about it?
   2. If no: Move on to the next question.

**ABC Problem Solving**

*The next set of questions are about the ABC problem-solving strategy you learned about in the CUIDA program.*

1. Did you try using the ABC strategy for one of your family member’s symptoms?

If yes:

- 1. For what symptom(s)?
  2. How did it go?
  3. What problems did you encounter with using the ABC strategy?

If no:

1. What is the reason?
2. How easy or hard is it to remember what “ABC” stands for? Please explain.
3. What did you think about the example in the videos of how a caregiver used the ABC strategy? The example of Doña Lucha and Don Juan.
   1. What did you like about the example?
   2. How can we make the example better?

**Caregiver’s Health & Wellbeing**

*Now we’ll move on to a different set of questions. Some caregivers have said that it would be helpful if the CUIDA program emphasized the caregiver’s own health and well-being. Caregivers said they needed to be well to take good care of another person.*

1. Do you relate to this? Why or why not?
2. Do you recall the times the last video in the program that talked about this topic? For example, it said, “We understand how hard it is to provide care to a family member with dementia. For that reason, it is very important to take good care of yourself too.”
   1. If yes: What did you think about it? What could have made it better?
   2. If no: Move on to the next question.

**Intervention Delivery**

*Now we’ll move on to the next set of questions related to using technology to participate in the CUIDA program.*

1. Did you prefer to sit down and watch the videos, or just listen to the audio while you were doing something else?
   1. Can you explain your preference?
2. How did you receive the videos? (e-mail, WhatsApp, text message, etc.)
3. What device did you use to watch the videos? (Cell phone, tablet, laptop, etc.)
   1. What problems did you encounter with viewing the videos?
4. We want to make these videos more available to family caregivers. What do you think are some ways that we can reach more *Latino* caregivers in particular?
5. Some caregivers have said that they shared the videos with their family members and friends. Did you ever share any of the videos with *your* family members or friends?

If yes:

- 1. Who did you share the video with?
  2. What motivated you to share the video?
  3. What did they think about the video?

If no:

1. Do you think other members of your family or any friends that help with caregiving might be interested in the CUIDA program? Why or why not?
2. What did you think about the design of the videos?
   1. What did you think about the:
      1. Colors?
      2. Audio/voice/narration?
      3. Pictures?
      4. Text?
3. How can we make the videos more appealing for family caregivers?

**Redcap Surveys**

*The last set of questions are about the surveys you completed online.*

1. What was your experience like completing the surveys online?
   1. What device did you use?
2. What problems did you encounter with completing the surveys online?

**Closing Question**

*We’re down to the final question before we end the interview.*

1. Is there anything else that we can do to make the CUIDA program better for Latino caregivers?

*****Stop recording*****

**Interview Guide (Spanish)**

*****Start recording*****

**Introducción**

*Antes de comenzar, me gustaría recordarle que su participación en este estudio es completamente voluntaria. En cualquier momento puede dejar de participar en la entrevista.*

*Como parte del estudio, usted utilizó el programa CUIDA durante los últimos 2 meses. El programa constaba de 7 videos y un libro de trabajo. El programa fue diseñado para ayudar a los cuidadores familiares, como usted, a aprender a manejar los síntomas de la demencia.*

*Como parte del estudio, también se le pidió que completara encuestas en línea.*

*Durante esta entrevista, quiero preguntarle sobre su experiencia con el programa CUIDA y con la realización de encuestas en línea. Le pedimos que brinde su opinión honesta, ya que nos ayudará a mejorar el programa para los cuidadores familiares que lo utilicen en el futuro. Estamos muy interesados en conocer sus ideas sobre cómo podemos mejorar el programa CUIDA para las familias latinas.*

*Dicho esto, ¿tiene alguna pregunta antes de que comencemos?*

**Preguntas Generales**

*Comenzaré haciendo preguntas generales sobre el programa CUIDA.*

1. ¿Qué piensa del programa?

a. ¿Qué tan útiles fueron los videos para usted?

b. ¿Qué tan útil fue el libro de trabajo para usted?

1. ¿Qué le gustó del programa?

a. ¿Qué le gustó más de los videos?

b. ¿Qué le gustó más del libro de trabajo?

1. ¿Hubo algo que faltara en el programa y le hubiera gustado ver?

a. ¿Cómo cree que podríamos mejorar los videos en el futuro?

b. ¿Cómo cree que podríamos mejorar el libro de trabajo en el futuro?

1. ¿Qué opinó sobre el idioma español en la intervención?

a. ¿Qué tan precisa fue la traducción al español?

b. ¿Qué tan fácil o difícil fue entender el español?

c. ¿Qué sugerencias tiene para mejorar el programa CUIDA en español?

**Educación sobre la demencia**

*Tal vez recuerdes que el primer video del programa proporcionaba información sobre la demencia. Hablaba sobre qué es la demencia, las etapas de la demencia y cómo, a medida que avanza, las personas tienen menos control sobre su comportamiento.*

1. ¿Qué opinó sobre este primer video?

a. ¿Qué información le resultó particularmente útil?

b. ¿Qué otros temas sobre la demencia le hubiera gustado discutir?

**Empatía**

*Algunos cuidadores han dicho que les gusta que se les recuerde que su familiar tiene una condición cerebral, que algunas de las cosas que su familiar dice o hace no son intencionales y que el cuidador debe tener más paciencia.*

1. ¿Se identifica con esto? ¿Por qué sí o por qué no?
2. ¿Recuerda momentos en que los videos tenían este tipo de recordatorios? Por ejemplo, uno de los videos decía: "Es importante recordar que los cambios que ocurren en su familiar no son intencionales".

a. Si respondió sí: ¿Qué piensas sobre esto?

b. Si respondió no: Pasemos a la siguiente pregunta.

**Las Tres Cs Para Prestación de Cuidados**

*Las siguiente preguntas son sobre la estrategia de “las tres Cs” para resolver problemas que aprendió en el programa CUIDA.*

1. ¿Intentó utilizar la estrategia “las tres Cs” para alguno de los síntomas de su familiar?

Si respondió sí:

a. ¿Para qué síntoma(s)?

b. ¿Cómo le fue?

c. ¿Qué problemas encontró al utilizar la estrategia “las tres Cs”?

Si respondió no:

a. ¿Cuál es la razón?

b. ¿Qué problemas encontró al utilizar la estrategia “las tres Cs”?

1. ¿Qué tan fácil o difícil es recordar qué significa "las tres Cs"? Explique, por favor.
2. ¿Qué piensa del ejemplo en los videos sobre cómo un cuidador utilizó la estrategia las tres Cs? El ejemplo de Doña Lucha y Don Juan.

a. ¿Qué le gustó del ejemplo?

b. ¿Cómo podemos mejorar el ejemplo?

**Salud y Bienestar del Cuidador**

*Ahora pasaremos a un conjunto diferente de preguntas. Algunos cuidadores han dicho que sería útil si el programa CUIDA enfatizara la salud y el bienestar del cuidador. Los cuidadores dijeron que necesitaban estar bien para cuidar bien a otra persona.*

1. ¿Se identifica con esto? ¿Por qué sí o por qué no?
2. ¿Recuerda los momentos en el último video del programa que hablaban sobre este tema? Por ejemplo, decía: " Entendemos lo difícil que es cuidar a un familiar con demencia. Por eso, es *muy* importante que usted también se cuide".

a. Si respondió sí: ¿Qué piense sobre esto? ¿Qué podría haberlo hecho mejor?

b. Si respondió no: Pasemos a la siguiente pregunta.

**Entrega de La Intervención**

*Ahora pasaremos al siguiente conjunto de preguntas relacionadas con el uso de la tecnología para participar en el programa CUIDA.*

1. ¿Prefirió sentarse a ver los videos o simplemente escuchar el audio mientras hacía otra cosa?

c. ¿Podría explicar su preferencia?

1. ¿Cómo recibió los videos? (correo electrónico, WhatsApp, mensaje de texto, etc.)
2. ¿Qué dispositivo utilizó para ver los videos? (teléfono celular, tableta, computadora portátil, etc.)

a. ¿Qué problemas encontró al ver los videos?

1. Queremos hacer estos videos más accesibles para los cuidadores familiares. ¿Qué cree que son algunas formas en las que podemos llegar a más cuidadores latinos en particular?
2. Algunos cuidadores han dicho que compartieron los videos con sus familiares y amigos. ¿Alguna vez compartió alguno de los videos con sus familiares o amigos?

Si respondió sí:

a. ¿Con quién compartió el video?

b. ¿Qué le motivó a compartir el video?

c. ¿Qué pensaron ellos sobre el video?

Si respondió no:

1. ¿Cree que otros miembros de su familia o amigos que ayudan con el cuidado podrían estar interesados en el programa CUIDA? ¿Por qué sí o por qué no?
2. ¿Qué te pareció sobre el diseño de los videos?

a. ¿Qué pensaste sobre:

i. Los colores?

ii. El audio/voz/narración?

iii. Las imágenes?

iv. El texto?

1. ¿Cómo podemos hacer que los videos sean más atractivos para los cuidadores familiares?

**Encuestas en Redcap**

*El último conjunto de preguntas se refiere a las encuestas que completó en línea.*

1. ¿Cómo fue su experiencia al completar las encuestas en línea?

a. ¿Qué dispositivo utilizó?

1. ¿Qué problemas encontró al completar las encuestas en línea?

**Pregunta Final**

*Llegamos a la última pregunta antes de terminar la entrevista.*

1. ¿Hay algo más que podemos hacer para mejorar el programa CUIDA para los cuidadores latinos?

*****Stop recording*****
